# Limited Trafficking of a Neurotropic Virus Through Inefficient Retrograde Axonal Transport and the Type I Interferon Response

**DOI:** 10.1371/journal.ppat.1000791

**Published:** 2010-03-05

**Authors:** Karen Z. Lancaster, Julie K. Pfeiffer

**Affiliations:** Department of Microbiology, University of Texas Southwestern Medical Center, Dallas, Texas, United States of America; University of Washington, United States of America

## Abstract

Poliovirus is an enteric virus that rarely invades the human central nervous system (CNS). To identify barriers limiting poliovirus spread from the periphery to CNS, we monitored trafficking of 10 marked viruses. After oral inoculation of susceptible mice, poliovirus was present in peripheral neurons, including vagus and sciatic nerves. To model viral trafficking in peripheral neurons, we intramuscularly injected mice with poliovirus, which follows a muscle–sciatic nerve–spinal cord–brain route. Only 20% of the poliovirus population successfully moved from muscle to brain, and three barriers limiting viral trafficking were identified. First, using light-sensitive viruses, we found limited viral replication in peripheral neurons. Second, retrograde axonal transport of poliovirus in peripheral neurons was inefficient; however, the efficiency was increased upon muscle damage, which also increased the transport efficiency of a non-viral neural tracer, wheat germ agglutinin. Third, using susceptible interferon (IFN) α/β receptor knockout mice, we demonstrated that the IFN response limited viral movement from the periphery to the brain. Surprisingly, the retrograde axonal transport barrier was equivalent in strength to the IFN barrier. Illustrating the importance of barriers created by the IFN response and inefficient axonal transport, IFN α/β receptor knockout mice with muscle damage permitted 80% of the viral population to access the brain, and succumbed to disease three times faster than mice with intact barriers. These results suggest that multiple separate barriers limit poliovirus trafficking from peripheral neurons to the CNS, possibly explaining the rare incidence of paralytic poliomyelitis. This study identifies inefficient axonal transport as a substantial barrier to poliovirus trafficking in peripheral neurons, which may limit CNS access for other viruses.

## Author Summary

Neurotropic viruses, including herpesviruses, rabies virus, and poliovirus, initiate infection in the periphery and can move through peripheral neurons to reach the central nervous system (CNS). Since peripheral neurons can be up to one meter long, inefficient neural transport could dramatically affect pathogenesis of neurotropic viruses. In this study, we used a novel viral “bar-coding” assay to quantify the efficiency of poliovirus transport from the periphery to the CNS using a mouse model. Only 20% of the poliovirus population successfully moved from the periphery to the CNS. We discovered that transport of poliovirus in peripheral neurons was very inefficient, and the innate immune response also limited viral movement. Surprisingly, the neural transport barrier was as strong as the innate immune response barrier. Importantly, by overcoming both the neural transport and innate immune barriers, 80% of the poliovirus population successfully moved from the periphery to the CNS, and mice succumbed to disease three times faster than mice with intact barriers. This study identifies inefficient neural transport as a substantial barrier to viral movement in peripheral neurons, which may limit CNS access for many viruses.

## Introduction

Many viruses are neurotropic, including West Nile virus, rabies virus, alpha herpesviruses, and poliovirus. To gain access and sustain infection in neurons, viruses must be able to efficiently traffic in axons, which can be up to one meter long. Therefore, viral trafficking in neurons requires an active transport system [1],[2]. Poliovirus is thought to enter neurons via receptor-mediated endocytosis at the neuromuscular junction, followed by endocytic transport from the nerve terminal to the cell body using the host retrograde axonal transport system. Poliovirus and some herpesviruses are thought to hijack the host transport machinery via Tctex-1, a component of the dynein light chain involved in retrograde axonal transport [3],[4].

Poliovirus is an enteric virus that rarely causes disease; however, in the pre-vaccine era, ∼1% of infected individuals developed paralytic poliomyelitis due to viral invasion of the central nervous system (CNS) and destruction of motor neurons. It is still unclear whether poliovirus accesses the CNS via blood or neural routes, but it has been shown that viremia is a prerequisite for CNS invasion of humans and non-human primates [5],[6]. In the 1990s, mice expressing the human poliovirus receptor (CD155/PVR) facilitated studies on poliovirus trafficking, although early models were limited in scope due to resistance of the mice to oral infection [7],[8]. Ohka et al. recently developed PVR mice lacking the interferon α/β receptor (IFNAR^−/−^), an important component of innate immunity, yielding PVR-IFNAR^−/−^ mice that are orally susceptible to poliovirus, and can be used to study viral dissemination following the natural route of infection [9]. While there is evidence for both blood and neural routes of poliovirus dissemination [10], recent *in vitro* studies with cultured neurons, and *in vivo* studies with PVR mice provide evidence for neural trafficking to the CNS [7],[11],[12],[13],[14]. It is thought that viremic blood seeds peripheral tissues, virus enters neurons of the peripheral nervous system (PNS) that innervate peripheral tissues, and virus traffics to the CNS using retrograde axonal transport.

Sciatic nerve models of poliovirus trafficking further support CNS access via a neural route following peripheral infection, because sciatic nerve transsection prevented disease in PVR mice intramuscularly injected with poliovirus [12],[14]. Similarly, sciatic nerve transsection prevented retrograde axonal transport of Theiler's virus, a picornavirus related to poliovirus [15]. Therefore, intramuscularly inoculated poliovirus traffics to the CNS in neurons via the sciatic nerve. The sciatic nerve contains a bundle of axons, each of which are single long cells that innervate the leg muscle and relay information from the periphery to their cell bodies in the spinal cord. Therefore, viral trafficking by this route requires viral uptake at the neuromuscular junction, active transport within the long axons of the sciatic nerve, viral release in the cell body within the spinal cord, and transport to the brain.

Here we use an artificial quasispecies to identify host barriers limiting viral trafficking from the periphery to the CNS. Previously, we uncovered a significant obstacle to viral trafficking between muscle and brain that severely bottlenecked the viral population [16],[17], and here we identify multiple barriers that contribute to this effect. By following viral population diversity, we discovered three distinct barriers the virus encounters between the periphery and the CNS: inefficient retrograde axonal transport in peripheral neurons, the type I interferon response, and limited viral replication in neurons of the PNS. To our knowledge, this is the first time that efficiency of viral retrograde axonal transport has been quantified, and identified as a major barrier limiting viral access to the CNS.

## Results

### Poliovirus Trafficking from Peripheral Tissues to the CNS is Limited by a Barrier Between the Peripheral and Central Nervous Systems

Previously, using 10 marked viruses, we identified host barriers that limit poliovirus trafficking from the gut to the CNS [16]. The marked viruses contain groups of 4–8 silent point mutations detectable by a hybridization-based assay, and constitute an artificial quasispecies that can be used to monitor viral population dynamics and identify host barriers that limit spread (Figure S1). Using this assay and another artificial quasispecies assay, a barrier was uncovered between a peripheral intramuscular injection site and the brain [16],[17]; however, the specific nature of this barrier was unknown. The goal of this study was to legitimize viral trafficking in PNS neurons as a potential route to the CNS following oral inoculation, and to identify the specific host barriers limiting viral trafficking from peripheral tissues to the CNS.

**Figure 1:**
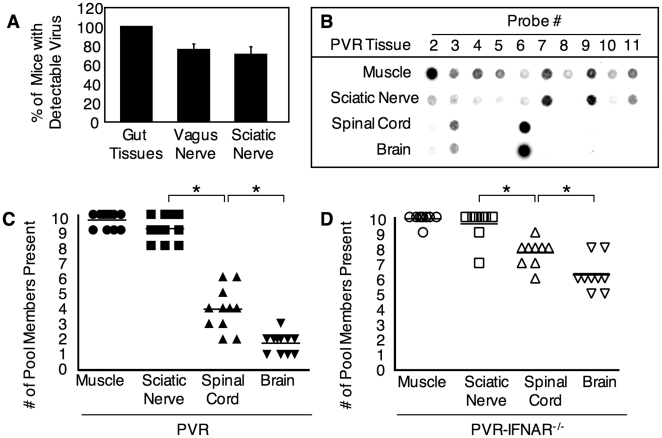
Identification of the barrier site between the periphery and CNS and the effect of type I interferon. (A) Detection of virus in peripheral nerves after oral infection. PVR-IFNAR^−/−^ mice were orally inoculated with 2×10^7^ PFU of poliovirus, tissues were harvested upon disease onset, and the percentage of mice with detectable virus was quantified by RT-PCR. Mean with SEM from 13–14 mice is shown; more details are shown in Figure S1. (B) A representative blot from the hybridization-based viral diversity assay. PVR mice were intramuscularly inoculated with 2×10^7^ PFU of the 10-marked viruses, tissue were collected upon disease onset (ranging from 2 to 6 days post-inoculation, see Figure 6), and viral pool members were detected with the viral diversity assay. Results from one representative mouse are shown. Viral population diversity in intramuscularly inoculated (C) PVR mice or (D) PVR-IFNAR^−/−^ mice. Mice were intramuscularly inoculated as in (B), and the number of pool members in each tissue for each mouse is shown. Horizontal lines represent mean diversity for each tissue. Asterisks denote statistically significant reductions in viral population diversity (Student's *t* test, p<0.01). Viral population diversity in PVR-IFNAR^−/−^ mice was significantly higher than in PVR mice across all tissues (p<0.0001, 2 way ANOVA).

To determine whether poliovirus is present in peripheral neurons following oral inoculation, orally susceptible PVR-IFNAR^−/−^ mice were orally inoculated with 2×10^7^ plaque forming units (PFU) of the 10-virus mixture, tissues were harvested upon disease onset, and viruses were detected by RT-PCR and the viral diversity assay. We monitored poliovirus in two peripheral nerves: the vagus nerve, which innervates multiple organs and is part of the enteric nervous system, and the sciatic nerve, which innervates leg muscle. Importantly, orally inoculated virus was detected in the vagus nerve in 76% of mice and in the sciatic nerve in 71% of mice (Figures 1A and S1).

**Figure 2:**
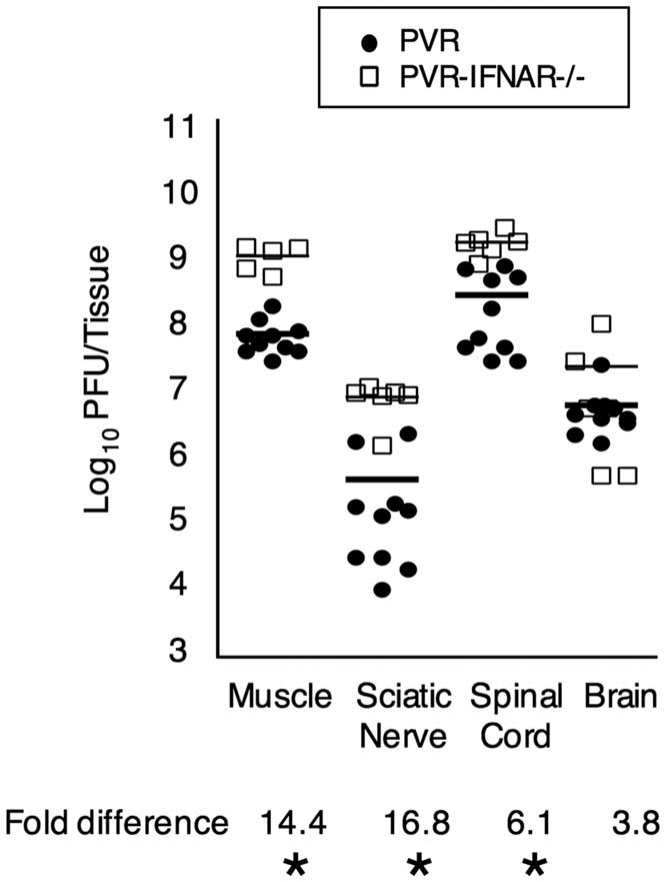
Viral titers from intramuscularly inoculated PVR and PVR-IFNAR^−/−^ mice. Mice were inoculated with 2×10^7^ PFU, tissues were harvested upon disease onset, and viral titers were determined by plaque assay. Titers from PVR mice are depicted as closed circles, and titers from PVR-IFNAR^−/−^ mice are depicted as open squares, with mean indicated by horizontal lines. The magnitude of the titer difference between PVR and PVR-IFNAR^−/−^ mice is indicated below each tissue, and asterisks denote statistically significant differences (p<0.0001, Student's *t* test).

Because poliovirus was detectable in sciatic nerve following oral inoculation, we used the sciatic nerve as a relevant model peripheral neuron to identify barriers contributing to the bottleneck effect encountered by the virus between peripheral organs and the CNS. PVR mice were intramuscularly inoculated with 2×10^7^ PFU of the 10-virus mixture, tissues were harvested upon disease onset, and viral population diversity was measured with the viral diversity assay (Figure 1B). Of the 10 original input viruses injected, we found an average of 9.5 viral pool members present in muscle, 9.0 in sciatic nerve, 4.2 in spinal cord, and 1.8 in brain (Figure 1C). Similar results were obtained for tissues harvested at an early time point, 30 hours post-infection (Figure S2). A dramatic decrease in the number of viral population members occurred between sciatic nerve and spinal cord, suggesting that the viral population encountered a major barrier between these sites. Importantly, viral titers from tissues do not reflect the dramatic bottleneck encountered by the viral population, because viral titers in spinal cord were 10,000-fold higher than viral titers in sciatic nerve (Figure 2). Therefore, the viral population was limited by a host barrier between the PNS and CNS, but robust replication occurred post-barrier in the CNS. These results uncovered barriers to viral trafficking that would have been masked by analyzing titer alone, and suggest that a significant barrier to viral CNS access occurs between the sciatic nerve and spinal cord.

We devised three hypotheses to explain the barrier between the PNS and CNS. First, the interferon response may limit peripheral replication, reducing the amount of virus in the periphery. Second, viral replication in peripheral neurons may be minimal, limiting the number of viruses entering the CNS. Third, retrograde axonal transport may be inefficient for poliovirus. We tested each of these hypotheses to dissect the mechanism of the PNS-to-CNS barrier.

### Host Innate Immunity Contributes to the Barrier

To determine whether the type I interferon response contributes to the sciatic-spinal cord barrier, we intramuscularly injected 2×10^7^ PFU of the 10-marked viruses into PVR-IFNAR^−/−^ mice, which lack the INFα/β receptor and are therefore deficient in generating a type I interferon response. Upon disease onset, tissues were harvested and viral population diversity was determined by the viral diversity assay. As shown in Figure 1D, 2.5-fold more viral pool members reached the brain in PVR-IFNAR^−/−^ mice than in PVR mice. Not surprisingly, viral titers were 4–17-fold higher in PVR-IFNAR^−/−^ mice than in PVR mice (Figure 2). Interestingly, the largest viral titer difference between PVR and PVR-IFNAR^−/−^ mice was in the periphery, suggesting that the interferon response limited viral trafficking by reducing replication in peripheral tissue. In fact, the difference between PVR and PVR-IFNAR^−/−^ viral titers in the brain was minimal (less than 4-fold) and not statistically significant. One interpretation of these results is that the type I interferon response exerts its effects in the periphery and may contribute to the viral bottleneck by limiting viral replication in peripheral tissues.

### Poliovirus Replication is Limited in Peripheral Neurons

Lack of replication in peripheral neurons could limit viral diversity and contribute to inefficient trafficking to the CNS. To quantify viral replication *in vivo*, we used light-sensitive polioviruses [16]. Poliovirus propagated in the presence of neutral red dye becomes light sensitive due to dye incorporation into the virion [18],[19],[20]. Exposure to light inactivates neutral red-containing virions, likely due to cross-linking of virion RNA; however, neutral red viruses maintain viability if not exposed to light. Upon uncoating, neutral red dye is diluted and viruses lose light sensitivity. Therefore, viral replication can be quantified by measuring the ratio of light-sensitive to light-insensitive virus. We have adapted this assay for *in vivo* studies by injecting mice with a pool of 10-marked neutral red viruses in the dark and comparing light-exposed versus non light-exposed tissue virus samples by viral titer analysis or the viral diversity assay.

**Figure 3:**
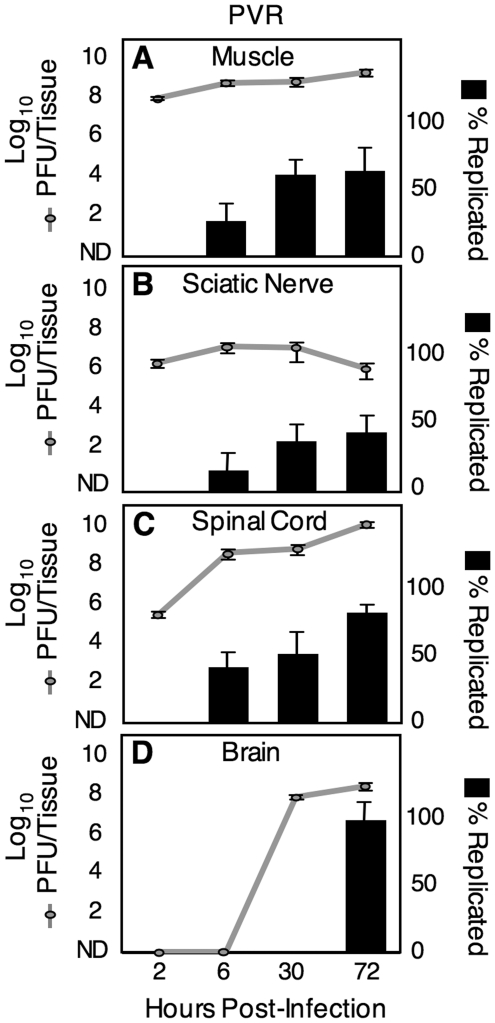
Viral replication kinetics. Mice were intramuscularly inoculated with 2×10^7^ PFU of light-sensitive/neutral red poliovirus in the dark, tissues were harvested at 2, 6, 30 and 72 hpi in the dark, and virus was extracted in the dark. Half of the virus sample for each tissue was exposed to light (inactivating non-replicated viruses, to quantify replicated viruses), and half was maintained in the dark (to quantify all viruses). Virus in light-exposed and non-exposed samples was quantified by plaque assay. Total titer represents titer from non-light-exposed sample and is expressed as PFU/tissue (grey lines). Therefore, the grey lines represent titer from inoculum virus plus replicated virus. The percent of replicated viruses was obtained by dividing the light-exposed titer by the non-light-exposed titer for each individual sample (black bars). Mean with SEM from 3–7 mice is shown.

First, we performed neutral red viral titer analysis to measure the kinetics of viral replication in various tissues along the route to the CNS. PVR mice were intramuscularly injected with 2×10^7^ PFU of the neutral red 10-virus pool, and muscle, sciatic nerve, spinal cord, and brain were harvested in the dark (using a red safety light) at 2, 6, 30 or 72 hours post infection (hpi). Tissues were processed in the dark, and samples of light-exposed virus and non-light exposed virus from each tissue were quantified by viral titer assay. Figure 3 shows the total titer (i.e., dark titer; grey lines), and the percent of virus that was replicated (i.e., light titer/dark titer x100; black bars) at each time point for different tissues, and the data indicate three key points. First, there was no evidence of viral replication in any tissue at 2 hpi, but there was evidence of viral replication in muscle, sciatic nerve and spinal cord at 6 hpi; therefore, viral replication is relatively fast *in vivo*. Second, virus was detectable in the spinal cord by 6 hpi, indicating that virus moves very quickly from the muscle injection site to the CNS, in agreement with previous work demonstrating viral movement by fast retrograde axonal transport [12]. The virus found in the spinal cord at 6 hpi was a mixture of replicated virus and non-replicated virus from the inoculum. Third, while titers in spinal cord increase over time by an average of 10,000-fold, titers in sciatic nerve remain relatively constant. Similarly, viral titers in muscle remain relatively constant despite viral replication at that site. Taken together these results suggest a model where virus in muscle is transported rapidly to the spinal cord via the sciatic nerve, but little or no replication occurs in the PNS (sciatic nerve); however, robust replication occurs in the CNS (spinal cord and brain).

**Figure 4:**
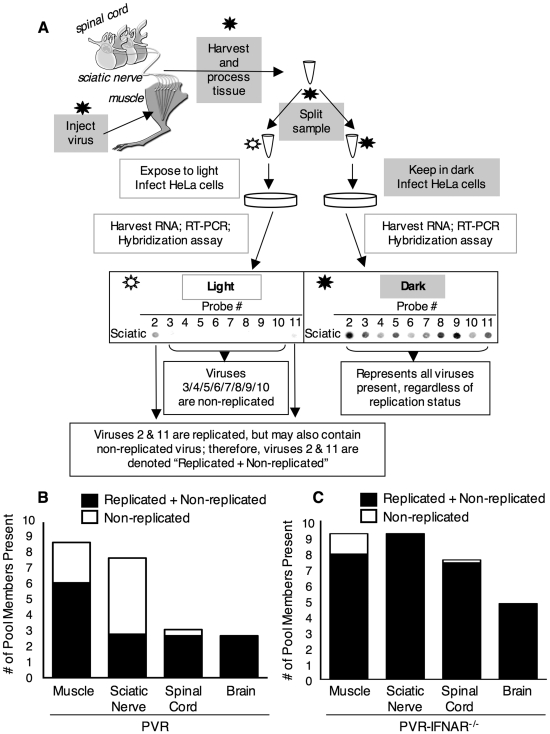
Replication status for individual viral population members. (A) Schematic of light-sensitive/neutral red poliovirus hybridization-based diversity assay for determining viral replication status. In the dark (using a red safety light; black star/grey boxes), the 10 marked viruses containing neutral red dye were intramuscularly injected into the left gastrocnemius muscle of PVR or PVR-IFNAR^−/−^ mice. At 72 hpi, tissues were harvested and processed in the dark. The viral sample for each individual tissue was split, and half of the sample was exposed to light to inactivate non-replicated virus (unfilled star/unfilled boxes), and the other half of the sample was kept in the dark. Both virus samples were amplified by a single cycle of replication in HeLa cells to expand surviving virus and decrease the prevalence of light-inactivated virus. Total RNA was extracted, and the tagged region of the viral genome was amplified by RT-PCR. DNA was then spotted on a membrane, and the viral diversity assay was performed. Signal for each virus from the light-exposed and non-light exposed samples was compared for each tissue; sciatic nerve from a representative mouse is shown as an example. Light-sensitive viruses were scored as ‘Non-replicated’ (in this case, viruses 3/4/5/6/7/8/9/10), and light-insensitive viruses were scored as ‘Replicated+Non-replicated’ (in this case, viruses 2 and 11) because they may contain a sub-population of non-replicated viruses, which would be masked by the signal from replicated viruses. Pooled results from each tissue for 10 PVR mice (B) and 5 PVR-IFNAR^−/−^ mice (C) are shown. Bars represent the mean diversity in each tissue, and the proportion of non-replicated virus is indicated (unfilled bar).

To further test the idea that poliovirus does not replicate in the sciatic nerve, we examined the replication status of individual viral population members using neutral red-virus in conjunction with the viral population diversity assay. PVR mice were intramuscularly injected with 2×10^7^ PFU of the 10-marked neutral red virus pool and tissues were harvested in the dark at 72 hpi, near the time of disease onset. After processing tissues in the dark, we divided the virus sample and exposed half to light and kept half in the dark. Light exposed and non-light exposed virus samples were amplified for a single cycle in HeLa cells to expand surviving viruses and minimize the prevalence of inactivated viruses. Replication status of tagged pool members was analyzed with the viral diversity assay by comparing the signal of every viral pool member on the “light” *vs.* “dark” blots (see Figure 4A). Light-sensitive viruses were scored as ‘non-replicated’, and light-insensitive viruses were scored as ‘replicated+non-replicated’ because they may contain a sub-population of non-replicated viruses, which would be masked by the signal from replicated viruses. ‘Non-replicated’ viruses consist exclusively of viruses that did not replicate while in the mouse, due to the absence of signal from the light-exposed sample. The results from 10 PVR mice are summarized in Figure 4B, which shows the overall number of viral pool members present in each tissue and the proportion of those pool members that were non-replicated viruses. In muscle, 30% of virus was non-replicated, in sciatic nerve, 64% of virus was non-replicated, in spinal cord, 13% of virus was non-replicated, and in brain, 0% of virus was non-replicated. These results reinforce the idea that robust replication occurs in CNS tissues, since 100% of brain viruses showed evidence of viral replication. Interestingly, even at 72 hpi, 30% of virus in muscle was non-replicated, indicating that this virus was stable and not cleared over a three day period, but remained viable since productive replication occurred in HeLa cells after tissue harvest. In sciatic nerve, the majority of viruses (64%) were non-replicated. Importantly, every single potentially replicated virus in sciatic nerve was also replicated in muscle (28/28), implying that “replicated” virus in sciatic nerve had undergone replication in muscle prior to sciatic nerve entry. Because the majority of viruses in sciatic nerve were non-replicated, and the minority of light-insensitive/replicated viruses had undergone replication in muscle, it is likely that poliovirus does not replicate in axons of the sciatic nerve. To test whether the interferon response limits viral replication, we repeated the experiment using PVR-IFNAR^−/−^ mice. Not surprisingly, we observed high percentages of replicated virus in all tissues (Figure 4C). These data, in conjunction with titer data from PVR-IFNAR^−/−^ mice (Figure 2B), imply that in the absence of the interferon response, viral replication in muscle was so robust that nearly all viruses replicated prior to entering the PNS. Taken together, these results suggest that poliovirus does not replicate in axons of peripheral neurons, rather, virus moves quickly from the peripheral injection site to the CNS, and once in the CNS, undergoes robust replication.

### Retrograde Axonal Transport of Poliovirus is Inefficient in Peripheral Neurons

**Figure 5:**
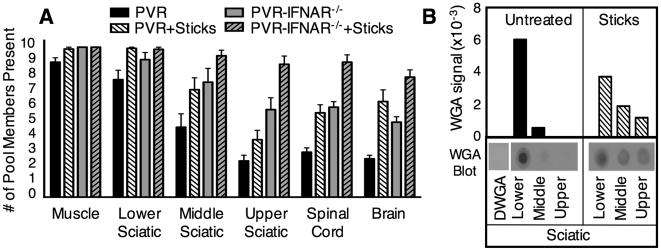
Quantification of retrograde axonal transport efficiency. (A) Efficiency of poliovirus transport and the effects of type I interferon and muscle damage. PVR and PVR-IFNAR^−/−^ mice were intramuscularly inoculated with 2×10^7^ PFU of the ten marked viruses, and were treated with or without needle sticks twice per day to induce muscle damage. Tissues were harvested at disease onset, and viral diversity was quantified. PVR mice, solid black bars; PVR mice with needle sticks, white hatched bars; PVR-IFNAR^−/−^ mice, grey bars; PVR-IFNAR^−/−^ mice with needle sticks, grey hatched bars. Results are expressed as mean with SEM, representing 4-13 mice per group. Statistically significant differences between groups are described in the text. (B) Efficiency of wheat germ agglutinin (WGA) transport and the effects of muscle damage. PVR mice were intramuscularly injected with 5 µg of WGA and treated with or without needle sticks. Tissues were harvested at 6 hours post-injection, and were processed for “dot-blot” protein immunoblot analysis. A WGA immunoblot, representative of four experiments, is shown under densitometry quantification (arbitrary units). Signal was normalized to sciatic nerve tissue from PVR mice not injected with WGA (denoted ΔWGA). Total WGA signal in the sciatic nerve represented approximately 1% of the WGA signal in muscle (data not shown).

Our data support previous work demonstrating that retrograde axonal transport of poliovirus is fast [4],[12],[21]; however, the efficiency of viral axonal transport has never been quantified. To determine whether inefficient retrograde axonal transport contributes to the barrier observed between sciatic nerve and spinal cord, we monitored viral population diversity during viral ascension of the sciatic nerve by harvesting segments of the nerve. PVR mice were intramuscularly injected with 2×10^7^ PFU of the 10-marked virus pool and viral diversity was quantified in the peripheral injection site (muscle), in three sections of the sciatic nerve (lower, middle, upper), in spinal cord, and in brain. As shown in Figure 5A (black bars), the lower section of the sciatic nerve contained an average of 8.3 pool members, middle sciatic contained 4.9 pool members, and upper sciatic contained 2.3 pool members. Therefore, the entire barrier between the sciatic nerve and spinal cord was due to loss of viral population members between the lower sciatic nerve and upper sciatic nerve. Poliovirus entry into the sciatic nerve at the neuromuscular junction was efficient, since 87% of pool members present in muscle were present in lower sciatic nerve; however, retrograde axonal transport was inefficient, since only 28% of pool members were successfully transported from lower sciatic nerve to upper sciatic nerve.

### Muscle Damage Increases Retrograde Axonal Transport and Poliovirus Trafficking to the CNS

While limited population diversity in the upper sciatic nerve suggests inefficient transport as a potential barrier to poliovirus trafficking, to formally demonstrate that transport inefficiency is the barrier, we determined whether increasing the efficiency of retrograde axonal transport would increase poliovirus population diversity in the CNS. Muscle damage via needle sticks is thought to enhance access to the CNS because Gromeier and Wimmer demonstrated enhanced poliovirus disease in mice subjected to needle sticks following intravenous inoculation [21]. To test whether needle sticks increase the efficiency of poliovirus retrograde axonal transport, PVR mice were intramuscularly injected with the 10-marked virus pool, and mice received needle sticks twice per day to induce muscle damage. Upon disease onset, tissues were harvested and the viral population diversity assay was performed (Figure 5). In mice that received needle sticks, the brain contained an average of 6.4 pool members, 3-fold more virus than untreated mice, suggesting that muscle damage increased poliovirus transport to the CNS (p<0.01, Students *t* test).

To verify that the muscle damage-mediated enhancement of poliovirus trafficking was due to increased efficiency of retrograde axonal transport, we monitored trafficking of a non-viral protein, wheat germ agglutinin (WGA), which is commonly used as a neural tracer [22],[23]. PVR mice were intramuscularly injected with 5 µg WGA, and treated with or without needle sticks. Tissues were harvested at 6 hours post injection, and WGA was quantified by immunoblotting (Figure 5B). In support of the idea that muscle damage increased retrograde axonal transport, WGA signal in middle and upper sciatic nerve was >3-fold higher in mice given needle sticks compared with untreated mice. Interestingly, the combined total of WGA signal for all sciatic nerve segments was nearly identical in both treatment groups, suggesting that WGA uptake at the neuromuscular junction was comparable; however, WGA was transported more efficiently in mice with muscle damage since more WGA was present in the middle and upper sections of the sciatic nerve. Therefore, both poliovirus and WGA have inefficient trafficking in the absence of muscle damage. Taken together, these data suggested that retrograde axonal transport of poliovirus is inefficient and constitutes a major barrier to viral access to the CNS, but that efficiency of transport to the CNS can be enhanced by muscle damage.

### Overcoming Host Barriers Facilitates Efficient Viral Trafficking to the CNS and Accelerates Disease Onset

**Figure 6:**
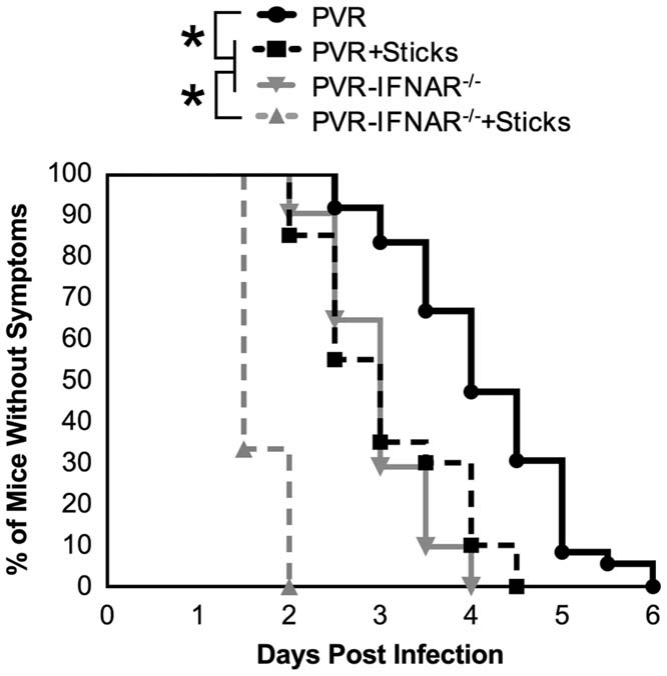
The effect of barriers on viral pathogenesis. PVR or PVR-IFNAR^−/−^ mice were intramuscularly inoculated with 2×10^7^ PFU of the 10-marked viruses, and were treated with or without needle sticks twice per day. Mice were euthanized upon disease onset; therefore, results are depicted as percent of mice without symptoms. Untreated PVR mice, solid black line with circles; PVR mice with needle sticks, dashed black line with squares; PVR-IFNAR^−/−^ mice, solid grey line with inverted triangle; PVR-IFNAR^−/−^ mice given needle sticks, grey dashed line with triangles. Data represent 12–36 mice per condition. Statistically significant differences between groups are indicated by asterisks (Mantel-Cox test, p<0.0001).

Having identified inefficient retrograde axonal transport and the interferon response as major barriers to viral trafficking, we sought to determine whether eliminating both barriers would facilitate efficient poliovirus trafficking to the CNS. PVR or PVR-IFNAR^−/−^ mice were intramuscularly injected with the 10-marked virus pool in the presence or absence of needle sticks, and population diversity was monitored. As expected, tissues from PVR-IFNAR^−/−^ mice contained significantly more population members in brain (2.5-fold) than PVR mice (p<0.01, Students *t* test) (Figure 5A). These numbers were comparable to the increased diversity in brain (3-fold) observed in PVR mice given needle sticks (p<0.001, Students *t* test). However, in PVR-IFNAR^−/−^ mice given needle sticks, sciatic nerve, spinal cord, and brain contained nearly all ten viruses (average of 9.4 in upper sciatic, 9.0 in spinal cord, and 8.0 pool members in brain), with significantly more viral pool members trafficking to the brain than in PVR-IFNAR^−/−^ mice or PVR mice given needle sticks (p<0.001, Students *t* test). Our results suggest that the type I interferon response and inefficient retrograde axonal transport may be separate barriers and that overcoming both barriers facilitated efficient viral trafficking to the CNS. This notion is further supported by the time of disease onset for each treatment group. As shown in Figure 6, using paralysis onset for 50% of mice per cohort as a measure of pathogenesis, untreated PVR mice developed disease on day 4.5 post infection, PVR mice given needle sticks and untreated PVR-IFNAR^−/−^ mice developed disease on day 3 post infection, and PVR-IFNAR^−/−^ mice given needle sticks developed disease on day 1.5 post infection. Therefore, overcoming one of the two barriers increased pathogenesis, as disease onset was 1.5-fold faster than mice with both barriers intact. Furthermore, eliminating two barriers dramatically enhanced pathogenesis, as disease onset was 3-fold faster than in untreated mice. Taken together, our results indicated that the type I interferon response and inefficient retrograde axonal transport are barriers of equivalent strength, and that these barriers reduce pathogenicity by limiting viral trafficking to the CNS.

## Discussion

Paralytic poliomyelitis due to viral CNS invasion and motor neuron destruction is very rare, occurring in less than 1% of unvaccinated individuals. A variety of hypotheses have been proposed to explain the rare poliovirus CNS invasion, ranging from fatigue to recent injury [21],[24],[25]. In this work, we identified three major barriers that may contribute to the rare incidence of paralytic poliomyelitis by limiting poliovirus trafficking from the periphery to the CNS: inefficient retrograde axonal transport, limited viral replication in the PNS, and the interferon response. Type I interferon has been shown to reduce replication of many viruses, and to limit the pathogenicity of neurotropic viruses [9],[26],[27],[28],[29],[30]. Perhaps predictably, we demonstrated that the type I interferon response can limit poliovirus dissemination by limiting replication in peripheral tissues, such as muscle.

Surprisingly, we identified inefficient retrograde axonal transport as a major barrier limiting poliovirus trafficking in PNS neurons and viral access to the CNS. In peripheral neurons, retrograde axonal transport of poliovirus and other viruses is very fast [1],[12],[31], and transport can be increased by muscle injury [14],[21]. While retrograde axonal transport may be fast, we demonstrate here that it is very inefficient for poliovirus, with only 28% of viral pool members successfully trafficking from lower to upper sciatic. By analogy, retrograde axonal transport of poliovirus can be thought of as a fast roller coaster without seatbelts, resulting in loss of passengers during the ride. The sciatic nerve consists of cells up to 5 cm long; therefore, active transport is required for viral trafficking. Although transport is inefficient, uptake of poliovirus at the neuromuscular junction is efficient, because 87% of pool members present in muscle were present in lower sciatic nerve. The inverse has been observed for neurotrophins, a class of host proteins that are transported by retrograde axonal transport [32],[33],[34],[35]. For neurotrophins, retrograde axonal transport is thought to be efficient and processive; however, neurotrophin cellular entry at the neuromuscular junction is quite inefficient [23],[36],[37]. Therefore, either viral transit mechanisms are not completely conserved with host transit mechanisms, or there are multiple host pathways that differ in retrograde axonal transport processivity [11]. It is also possible that poliovirus overwhelms the transport system or is degraded during retrograde axonal transport, thus explaining inefficient transport despite efficient uptake at the neuromuscular junction. Alternatively, retrograde axonal transport of host cargo may be as inefficient as poliovirus transport.

While retrograde axonal transport of poliovirus in peripheral neurons was inefficient, the efficiency increased upon muscle damage. Gromeier and Wimmer suggested that muscle damage enhances poliovirus CNS access and contributes to some cases of paralytic poliomyelitis [14],[21], and nerve injury is known to increase retrograde axonal transport of neurotrophins [38]. Additionally, muscle injury induces inflammation, which may also impact viral trafficking. Provocation poliomyelitis occurs when physical trauma near the time of poliovirus infection coincides with increased incidence of paralytic poliomyelitis. This effect was observed during the Cutter incident, where batches of incompletely inactivated poliovirus vaccine caused paralysis preferentially in the inoculated limb [39],[40]. Additional cases of paralytic poliomyelitis occurred when the attenuated oral polio vaccine was administered near the time of multiple unrelated intramuscular injections [41]. Our data demonstrating that muscle damage increases the efficiency of retrograde axonal transport may provide the mechanism for the increased incidence of paralytic poliomyelitis following muscle damage.

Several viruses traffic in PNS neurons to reach the CNS. For example, reovirus can traffic to the CNS via the vagus and sciatic nerves, and alpha herpesviruses traffic to the CNS in PNS neurons [42],[43],[44],[45],[46]. Our results suggest that after oral infection, poliovirus may traffic through PNS neurons to the CNS because orally inoculated poliovirus was detected in peripheral neurons (vagus and sciatic). We also found that the viral pool members present in sciatic and vagus nerves were highly bottlenecked, and matched brain virus 63% of the time (Figure S1 and data not shown), suggesting that transport of virus from PNS neurons to the CNS may occur after natural oral infection.

In addition to inefficient retrograde axonal transport in neurons, we found that poliovirus replication was limited in peripheral neurons. Using light sensitive viruses, we found no evidence of viral replication in the sciatic nerve despite robust replication in the CNS. These results are supported by data from Ohka et al., showing intact 160S virions in sciatic nerve [12]. Perhaps it is not surprising that viral replication is limited in peripheral neurons, since substrates required for viral replication are likely to be limited in long axons, and viruses may reside in endosomes during the entire axon transport period. Nonetheless, if virions or virion-containing endosomes disassociate from the retrograde axonal transport machinery, viral replication may be impossible within the axon.

Taken together, our results support the neural route as a major pathway of poliovirus trafficking to the CNS in mice; however, trafficking in neurons is difficult due to inefficient retrograde axonal transport. We propose that PNS barriers contribute to the low incidence of paralytic poliomyelitis in humans, and may contribute to inefficient trafficking of other neurotropic viruses.

## Materials and Methods

### Ethics Statement

All animals were handled in strict accordance with good animal practice as defined by the relevant national and/or local animal welfare bodies, and all animal work was approved by the UT Southwestern Medical Center IACUC.

### Viruses and Cells

Virulent Mahoney type 1 poliovirus was propagated and titered in HeLa cells as previously described [16],[47]. The ten marked viruses for the viral diversity assay each contain groups of 4–8 silent point mutations that allow specific probe annealing following RT-PCR and dot blotting (see Figure S1 and [16] for more details). These viruses exhibit no detectable fitness differences [16]. Light sensitive poliovirus was prepared and analyzed as previously described [16],[18],[19],[20]. Briefly, HeLa cells were infected with each marked virus in the presence of 10 µg/ml neutral red dye. Work with neutral red viruses was preformed in the dark, using a red photography light. Inactivation of neutral red viruses was achieved by exposure to a fluorescent light for 10 min. Samples were processed in the dark and supernatant from each tissue was divided in half (half was then exposed to light and the other half was always kept in the dark). For titer analysis, the ratio of PFU in light exposed versus non-light exposed samples were compared to determine the percent replicated virus [16]. The ratio of light-insensitive to light-sensitive PFU in the neutral red poliovirus stock was 1 to 1,270,000 [16]. Based on previous work and this study, we routinely detect 100% of in vitro and in vivo replicated viruses using this assay (data not shown) [16].

### Mouse Experiments

C57/BL6 mice expressing the human poliovirus receptor (CD155/PVR, called PVR) and C57/BL6 PVR mice deficient in the interferon-α/β receptor (called PVR-IFNAR^−/−^ mice) were a generous gift from S.Koike (Tokyo, Japan) [7],[29]. Oral inoculations were performed by pipetting 2×10^7^ total PFU of an equal mixture of all 10 viruses in 15 µl volume into the mouth [16]. Inoculum was prepared by mixing 2×10^6^ PFU of each virus per mouse (according to viral titer assay), and a large cocktail was prepared for each experiment such that all animals received the same mixture. For intramuscular injections, 2×10^6^ PFU of each marked virus (2×10^7^ total PFU in 50 µl) was injected into the lower left gastrocnemius muscle [16]. Needle sticks were given by inserting a 28-gauge needle into the leg 4 or 5 times twice daily [21]. For all poliovirus experiments, mice were monitored twice a day (at ∼10–14 hour intervals) and euthanized at the first sign of disease, which is typically paralysis of one hind limb. Upon onset of symptoms, mice fail to recover and typically succumb to disease within 12 hours (data not shown). Therefore, time of disease onset correlates with time of death, and can be used as a more humane alternative to death as an endpoint.

### Tissue Harvest and Processing

Whole sciatic nerve was removed by lifting the biceps femoris and removing the nerve segment between the spine and ankle. The nerve was then sectioned into three equal pieces to generate upper, middle and lower sciatic sections. The vagus nerve was removed as a segment from the heart-lung junction to the diaphragm. Muscle included all non-bone tissue below the hip. Tissues (whole spine, brain, stomach, small intestine, colon) were weighed and resuspended in three volumes PBS+ (1× PBS with 100 µg/ml MgCl_2_ and CaCl_2_), and homogenized in liquid nitrogen with a mortar and pestle [17] or with a Bullet Blender tissue homogenizer (Next Advanced Inc, Averill Park, NY) as per manufacturers instructions, followed by freeze-thawing three times to release virus and chloroform extraction of gut samples to inactivate bacteria [16]. Vagus and sciatic nerve tissue were dounce homogenized. All samples and tissues were stored at −80°C.

### Hybridization-Based Viral Population Diversity Assay

Detection of the 10 marked polioviruses was performed as previously described [16]. Briefly, viruses from homogenized tissues with low viral titers (stomach, colon, small intestine, vagus, and sciatic nerve) were amplified in HeLa cells, followed by TRIZOL (Invitrogen, Carlsbad, CA) extraction of RNA. Tissues with high viral titers (brain, spine, muscle) were directly extracted with TRIZOL because there was no difference between results from amplifying viruses in HeLa cells and direct TRIZOL extraction of high titer tissues (data not shown). RT-PCR for the tagged region of the virus was performed as previously described [16]. After blotting equivalent concentrations of PCR products on Hybond N+ membranes (GE Healthcare, Buckinghamshire, UK) individual membranes were pre-hybridized and hybridized at 59°C. Primers specific for each of the 10 viruses were kinased with [γ-^32^P]ATP to serve as probes [16]. Following hybridization, membranes were exposed to PhosphorImager screens and specific signal was determined by normalizing blots to perfectly matched and mismatched control PCR product dots and image intensity was uniformly adjusted until mismatched sample was no longer visible in order to eliminate low low-level cross reactive signal (see [16] for more details). For the neutral red diversity assay in Figure 4, signal from light exposed samples was compared to signal from non-light exposed samples from the same tissue. Any viral pool members present only in the dark sample were scored as ‘non-replicated’ virus, and viral pool members present in both the dark and the light sample were scored as ‘replicated+non-replicated’ (see Figure 4 for more details).

### Wheat Germ Agglutinin Experiments

Tissues were collected from mice 6 hours after injecting 5 µg WGA into the lower gastrocnemius muscle. Muscle was weighed and resuspended in 2 volumes of RIPA buffer (10 mM Tris,150 mM NaCl, 0.02% NaN_3_, 1% Na-deoxycholate, 1% Triton X-100, 0.1% SDS) and sciatic nerve was resuspended in 200 µl of RIPA buffer. Samples were then homogenized with the Bullet Blender, and 10 µl/ml of a protease inhibitor cocktail (Sigma, St Louis, MO) and 10 µl/ml of a phosphatase inhibitor (Calbiochem, San Diego, CA) were added to the supernatants. Dot blot westerns were performed in place of typical gel-based westerns due to multimerization of WGA; therefore, five microliters of each sample was pipetted directly onto a nitrocellulose membrane (GE Water & Process Technologies), which was probed with rabbit anti-lectin (triticum vulgaris) primary antibody (Sigma, St Louis, MO) and goat anti-rabbit HRP secondary antibody. Signal was visualized with ECL reagent (GE Healthcare, Buckinghamshire, UK) and quantified by densitometry [48]. Specific WGA signal was distinguished from background by normalizing to a sciatic nerve sample that was not exposed to WGA. WGA signal was within the linear range of detection based on loading and quantification of purified WGA dilutions (data not shown).

## Supporting Information

Figure S1 Artificial quasispecies system and poliovirus population dynamics following oral inoculation of PVR-IFNAR^−/−^ mice. (A) Sequences of marked viruses, showing silent mutations (bold underline) in the poliovirus genome. (B) A representative blot from an orally inoculated PVR-IFNAR^−/−^ mouse, and (C) results from all orally inoculated PVR-IFNAR^−/−^ mice. Mice were orally inoculated with 2×10^7^ PFU of the 10-marked viruses, and tissues were harvested upon disease onset. Horizontal lines represent mean diversity for each tissue.
(2.37 MB TIF)

Figure S2 Poliovirus population diversity in tissues from intramuscularly injected PVR mice. PVR mice were intramuscularly injected with 2×10^7^ PFU of the 10-virus mixture, and tissues were harvested at an early time point, 30 hours post-infection (hpi), or a late time point, 72 hpi (near the time of disease onset). Viral population diversity was determined by the hybridization assay, and results from 3-8 mice per group are shown with SEM.
(1.63 MB TIF)
