# Supplementary figures and images for: Limited Trafficking of a Neurotropic Virus Through Inefficient Retrograde Axonal Transport and the Type I Interferon Response
Source: PLoS Pathog. 2010 Mar 5;6(3):e1000791. doi: 10.1371/journal.ppat.1000791 (PMC2832671; doi:10.1371/journal.ppat.1000791)

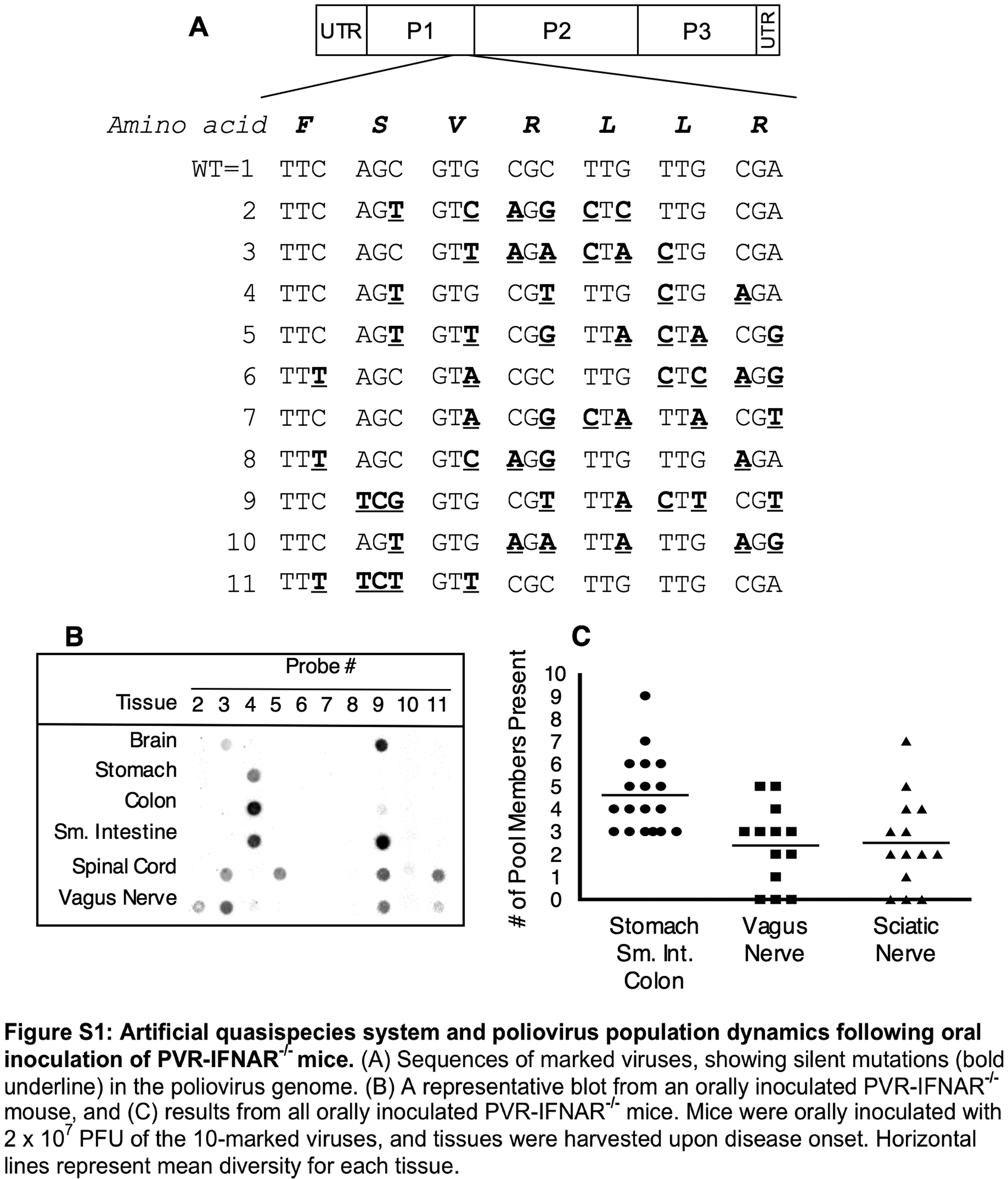

Supplement: Figure S1 — Artificial quasispecies system and poliovirus population dynamics following oral inoculation of PVR-IFNAR−/− mice. (A) Sequences of marked viruses, showing silent mutations (bold underline) in the poliovirus genome. (B) A representative blot from an orally inoculated PVR-IFNAR−/− mouse, and (C) results from all orally inoculated PVR-IFNAR−/− mice. Mice were orally inoculated with 2×10^7^ PFU of the 10-marked viruses, and tissues were harvested upon disease onset. Horizontal lines represent mean diversity for each tissue. (2.37 MB TIF) [file ppat.1000791.s001.tif]

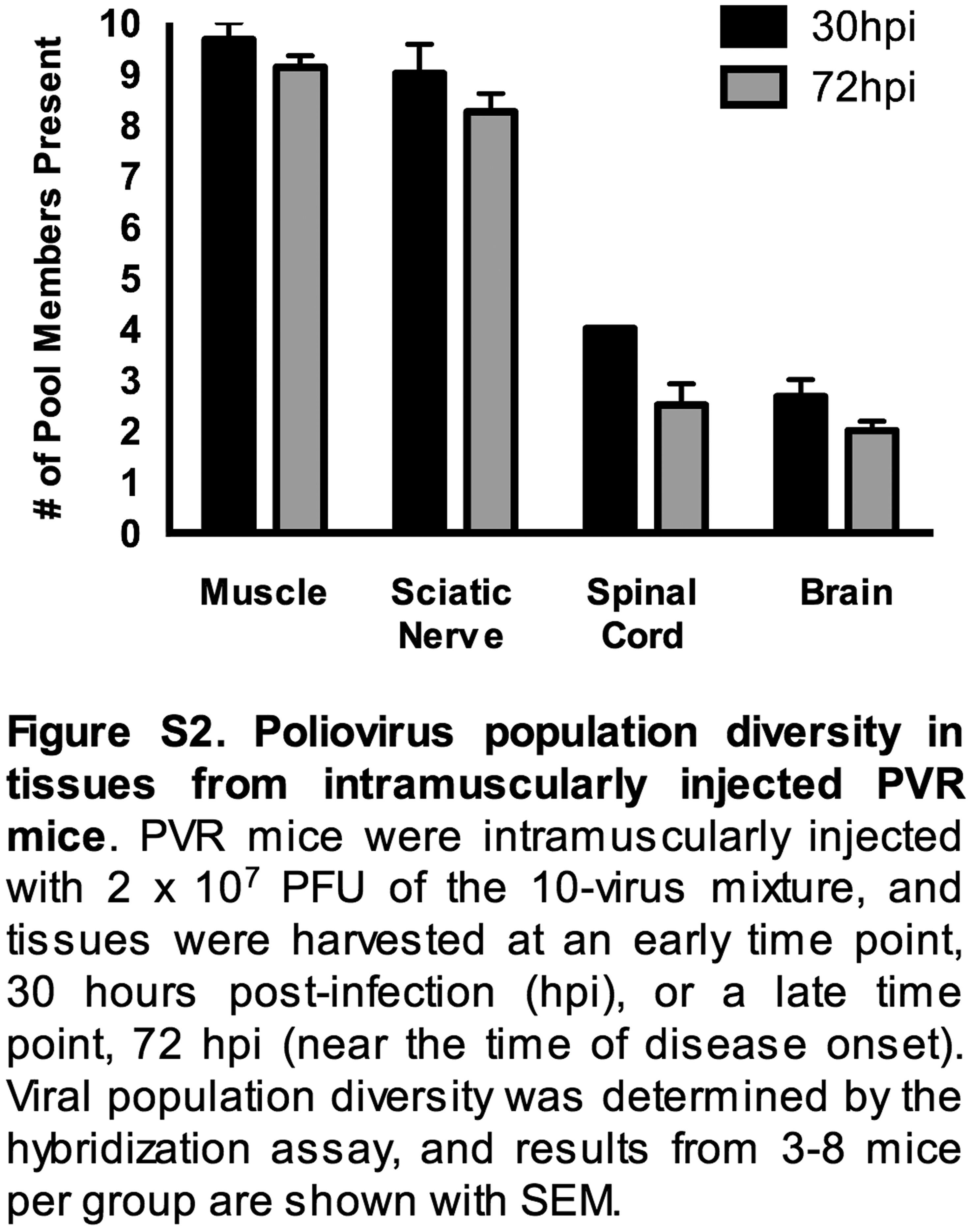

Supplement: Figure S2 — Poliovirus population diversity in tissues from intramuscularly injected PVR mice. PVR mice were intramuscularly injected with 2×10^7^ PFU of the 10-virus mixture, and tissues were harvested at an early time point, 30 hours post-infection (hpi), or a late time point, 72 hpi (near the time of disease onset). Viral population diversity was determined by the hybridization assay, and results from 3-8 mice per group are shown with SEM. (1.63 MB TIF) [file ppat.1000791.s002.tif]
